# Supplementary material for: P-TRAP: a Panicle Trait Phenotyping tool
Source: BMC Plant Biol. 2013 Aug 29;13:122. doi: 10.1186/1471-2229-13-122 (PMC3848748; doi:10.1186/1471-2229-13-122)
Supplement: Additional file 7 — P-TRAP source code. The Netbeans project that contains the source code of the application. [file 1471-2229-13-122-S7.zip › javadoc.zip!/javadoc/org-bioalg-reportprocessor/index-all.html]

Index (ReportProcessor)


JavaScript is disabled on your browser.


- Package
- Class
- Tree
- Deprecated
- Index
- Help

*org.bioalg.reportprocessor 1.0*

- Prev
- Next

- Frames
- No Frames

- All Classes

G O R S 


## G

generateReport() - Method in class org.bioalg.reportprocessor.Report
:   Collects the results and generate a master report

generateReport2() - Method in class org.bioalg.reportprocessor.Report
:   Generate a detailed report of panicle and grain.

generateReport3(String) - Method in class org.bioalg.reportprocessor.Report
:   Generate a detailed report of the panicle and grains.

getGraphModel() - Method in class org.bioalg.reportprocessor.Report


getImagefile() - Method in class org.bioalg.reportprocessor.Report


getPanicleRadius(Point, int) - Method in class org.bioalg.reportprocessor.Report
:   Find the panicle diameter by using the Euclidian Distance Map within a
    search range centered at the first generating point (center parameter).

GetParticlesReport() - Method in class org.bioalg.reportprocessor.Report
:   Create and return a string report of the grain traits only.

getPm() - Method in class org.bioalg.reportprocessor.Report


GetSumParticlesReport() - Method in class org.bioalg.reportprocessor.Report
:   Generate a summary report for the grains.


## O

org.bioalg.reportprocessor - package org.bioalg.reportprocessor


## R

Report - Class in org.bioalg.reportprocessor
:   The reports generator class.

Report(GraphModel) - Constructor for class org.bioalg.reportprocessor.Report


Report() - Constructor for class org.bioalg.reportprocessor.Report


Report(ParticlesModel) - Constructor for class org.bioalg.reportprocessor.Report


Report(GraphModel, ParticlesModel) - Constructor for class org.bioalg.reportprocessor.Report


Report(Element, Element) - Constructor for class org.bioalg.reportprocessor.Report
:   Constructor by using xml files for the graph and particles models


## S

seperateJunctions() - Method in class org.bioalg.reportprocessor.Report


setGm(GraphModel) - Method in class org.bioalg.reportprocessor.Report


setImagefile(File) - Method in class org.bioalg.reportprocessor.Report


setPm(ParticlesModel) - Method in class org.bioalg.reportprocessor.Report

G O R S

- Package
- Class
- Tree
- Deprecated
- Index
- Help

*org.bioalg.reportprocessor 1.0*

- Prev
- Next

- Frames
- No Frames

- All Classes
